# MEMS-Scanner Testbench for High Field of View LiDAR Applications

**DOI:** 10.3390/s22010039

**Published:** 2021-12-22

**Authors:** Valentin Baier, Michael Schardt, Maximilian Fink, Martin Jakobi, Alexander W. Koch

**Affiliations:** 1 Institute for Measurement Systems and Sensor Technology, Technical University of Munich, 80333 Munich, Germany; max.fink@tum.de (M.F.); m.jakobi@tum.de (M.J.); a.w.koch@tum.de (A.W.K.); 2 Optics Department, Blickfeld GmbH, 80339 Munich, Germany; michael.schardt@blickfeld.com

**Keywords:** MEMS, LiDAR, scanning, testbench

## Abstract

LiDAR sensors are a key technology for enabling safe autonomous cars. For highway applications, such systems must have a long range, and the covered field of view (FoV) of >45° must be scanned with resolutions higher than 0.1°. These specifications can be met by modern MEMS scanners, which are chosen for their robustness and scalability. For the automotive market, these sensors, and especially the scanners within, must be tested to the highest standards. We propose a novel measurement setup for characterizing and validating these kinds of scanners based on a position-sensitive detector (PSD) by imaging a deflected laser beam from a diffuser screen onto the PSD. A so-called ray trace shifting technique (RTST) was used to minimize manual calibration effort, to reduce external mounting errors, and to enable dynamical one-shot measurements of the scanner’s steering angle over large FoVs. This paper describes the overall setup and the calibration method according to a standard camera calibration. We further show the setup’s capabilities by validating it with a statically set rotating stage and a dynamically oscillating MEMS scanner. The setup was found to be capable of measuring LiDAR MEMS scanners with a maximum FoV of 47° dynamically, with an uncertainty of less than 1%.

## 1. Introduction

Autonomous vehicles are the next step in transportation. To achieve this goal, cars must be equipped with sensors that perceive the environment in detail and accurately. Modern setups ensure safe driving by combining perception sensors such as cameras, radars, and especially LiDARs. LiDAR sensors in particular are one of the key enablers of automotive driving since their output provides direct distance information on every object in the scanned field of view (FoV). LiDAR (short for light detection and ranging) sensors are most commonly used as time-of-flight sensors. They can determine the distance to any object they face by measuring the time that an emitted laser pulse takes to arrive at an object, be scattered by it, and finally return to the sensor and be detected. Modern LiDARs differentiate themselves mainly in their approach to scanning a scene. Depending on the application and light source, capturing the scene can be achieved without a scanner (FLASH-LiDAR), or a combination of one-dimensional scanning and a one-dimensional sensor, or two-dimensional scanning. While, mechanically, one-dimensional scanning systems were the first introduced to this market [1], many big companies focus on solid-state technology such as micro-electro-mechanical systems (MEMS) to enable steering in two dimensions. The main reason for using MEMS is the scalability of the production and hence the reduction in price [2].

The requirements for LiDAR MEMS scanners are derived from automotive-use cases, one of which is highway driving. Highway scenarios are crucial since high vehicle speeds lead to long breaking times, which means that even small obstacles can create deadly accidents. Four main parameters must to be addressed for this use case in general: range, FoV, resolution, and frame rate. For the sake of simplification, the following description focuses on 2D-scanning LiDAR sensors. With respect to range, the most substantial influence comes from the detecting aperture, which itself is highly affected by the optical size of the deflecting surface of the scanner. Thus, MEMS scanners with large apertures are needed [3]. The FoV of a scanning LiDAR is solely created by the used scanners as they steer the illumination and detection cone into the desired direction. The FoV is defined as the maximum solid angle extent that can be covered by the sensor. For the depicted scenario of highway driving, large horizontal FoVs of around 40° to 60° are geometrically required to detect the full road ahead when including mounting tolerances and different road scenarios. Many companies have already committed themselves to buildings sensors with a horizontal FoV of 60° [4]. The vertical FoV is typically specified as ca. 20–30° [4,5]. The horizontal value can be directly projected and used as a specification for the scanning range of the MEMS. Inside the covered FoV, the density of capture is defined by the resolution of the pattern with which the laser is fired while scanning. It enables the differentiation of small objects over vast distances and is typically specified as ca. 0.1° [4,5]. Achieving this requires a dynamic characterization of MEMS scanners with low uncertainty. Since pulsing on a sinusoidal oscillation to achieve the resolution is directly proportional to speed and thus to the maximum steering angle (FoV), an accurate measurement of the FoV with a relative uncertainty of 1% is sufficient for this requirement. In operation, this ensures that individual acquisitions are spatially grouped with the same relative uncertainty. This generates a consistent pulse pattern without gaps. Lastly, a high frame rate is needed to increase the confidence in detected objects. This figure of merit describes the rate at which the full FoV is captured exactly once. In general, it can be said that the more optical channels, the higher the oscillation frequencies of the scanner, the lower the vertical FoV, and the smaller the vertical resolution, the higher the frame rate. Thus, scanners oscillate with frequencies of around 250 Hz, enabling high frame rates above 10 Hz–20 Hz [4,5]. Dynamically measuring or characterizing the steering angles of such scanners with these merits in mind is a highly difficult task, especially because of the required accuracy and the overall angular range.

Calibration of 3D LiDARs has been performed in the past, mainly focusing on the calibration of the entire device and the effects on mounting [6,7]. This ensures that the position of detected objects is correct compared to the coordinate system of the car. The procedure is directly influenced by the specific mounting pose and is especially important whenever multiple sensors are fused. Diverging from this kind of calibration, a different approach is the characterization of the individual scanners within a sensor. As they are deeply integrated into generating the desired capturing pattern in a point cloud, it is enormously important to characterize scanners with a high degree of confidence. The following paragraphs cover possible setups and techniques to characterize such MEMS scanners.

Measuring steering angles can—in its easiest form—be achieved simply by using a laser that is steered by the mirror of the scanner onto a projection plane, which was used as a validation technique by Fujita et al. [8]. Using trigonometric equations, a displacement of the projected spot gives the steered angle. When dynamically determining the position of a laser spot, the most accurate and most used sensor is a Position-Sensitive Device (PSD). Available as two-dimensional sensors with sizes up to 20 mm × 20 mm, they are capable of measuring the laser spot’s weighted center of intensity with micrometer uncertainties. These sensors have been used in the past for similar applications such as fast autofocus inspections [9] as well as angle measurements of the relative story displacement [10]. The dynamic potential of the PSD has also been observed in the past [11], which showed that dynamic measurements with speeds up to 104.72 m/s (=ca. 10.5 mm/100 μs) are possible with a PSD.

Measurements of mirror-inclination angles or MEMS steering angles with PSDs have been performed by [8,12,13,14]. Nishibori and Nishibori [9] focused on a static measurement in a range of ±8° with a measurement uncertainty of 0.5°. Fujita et al. [8] designed a controlling system with an rms error of 0.21° in an angular range between ±6° for a two-dimensional MEMS mirror and a holed PSD, which showed that the dynamic characterization of MEMS with PSDs is possible. A third study focused on the evaluation of small, dynamic angular rotations with different mirror speeds which led to dynamic measurements with a measurement uncertainty of 1.9 arcseconds (≈0.032°) in a range of 400 arcseconds (≈6.7°) [13]. The most recent results are from Han Woong Yoo et al. [14],who described a MEMS-scanner test bench that is able to dynamically measure steering angles of up to 15° with a measurement uncertainty of 0.026°, by using adjustment mounts and a stage shifting technique.

All described methods for measuring steering angles of mirrors are insufficient for characterizing MEMS scanners for LiDAR applications because none fulfills the required combination of measuring a large FoV of at least 40° dynamically with low measurement uncertainties. Furthermore, current setups require many accurate calibration steps. To achieve the derived merits, we propose a novel measurement technique of characterizing large-FoV MEMS scanners by using a projection screen and a so-called ray trace shifting technique of a PSD camera setup. We further extend the shifting technique described in [14] to reduce the necessary alignment steps of the scanner and enable a fast characterization procedure. The system was calibrated using a translation stage and the camera model of Zhang [15]. The capability of the built measurement system was then verified statically with a rotation stage. A final validation step compared the measured FoV for three different driving amplitudes of a MEMS scanner with a reference to quantify the results.

## 2. Setup and Methods

This chapter explains the components and the general setup of the system in detail. It further describes how the setup is calibrated and validated. Finally, it provides the equations that are necessary to derive the steering angle of the MEMS scanner from the measured sensor data.

### 2.1. General Setup Description

**Figure 1:**
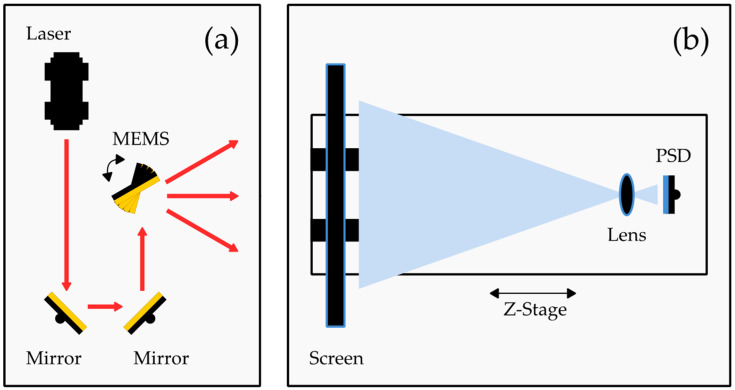
General setup of the testbench: (**a**) emission path; (**b**) detection path.

The proposed setup captures a projected laser spot on an optical diffuser screen with a calibrated imaging system consisting of a PSD lens combination. The steering angle of the MEMS scanner can be derived from the knowledge of the imaging system and the distance between the scanner and the diffuser screen. This enables the measurement of large steering angles, high dynamic bandwidth, and small measurement uncertainties thanks to the quality of the PSD as a detector. A schematic of the setup for characterizing MEMS-based LiDAR scanners is depicted in Figure 1.

As can be seen in Figure 1, the setup consists of two main parts: the emission path (a) and the detection path (b). The emission path comprises a laser, two mirrors, and the MEMS scanner. The laser, a 200 mW CW laser (RLDH980-200-3, Roithner Lasertechnik GmbH, Vienna, Austria) with a center wavelength of 980 nm and a collimating lens, generates a laser spot with a diameter of approx. 8 mm at the projection screen. The mirrors adjust the beam to be perpendicularly aligned in regards to the base plate. The laser beam is steered by the MEMS scanner under test, which is a MEMS scanning module similar to [16] with a variable scanning amplitude. Here, the driving amplitude is set to reach ca. 50°. The steered laser beam hits the screen, where a projection figure is created. For a perfectly sinusoidal oscillating scanner, a straight, horizontal line is created as a projection figure.

The screen itself, which is 23 mm to 48 mm away from the MEMS scanner, is a transmissive glass diffuser (Broadband Hybrid Diffusers, #36619, Edmund Optics Inc, Barrington, NJ, United States) with a Lambertian scattering distribution. The scattered light from the diffuser is captured by an aspheric lens (A240TM-B, Thorlabs GmbH, Munich, Germany) with a focal length of 8 mm, which creates an image of the projection figure on a two-dimensional PSD sensor (PDP90A, Thorlabs GmbH, Munich, Bavaria, Germany) with a total sensing area of 9.1 mm × 9.1 mm and a bandwidth of 16 kHz. The PSD is connected to an oscilloscope (RTB2004, Rohde & Schwarz GmbH & Co. KG, Munich, Germany), which is triggered by the control unit of the MEMS scanner. The oscilloscope captures the PSD data and saves them for postprocessing. The measured voltages are converted into sensor positions according to the following Equation (1) from [17]. The symbols are slightly altered to be consistent throughout.

(1) $$\left[\begin{array}{c} u_{d}\\ v_{d} \end{array}\right]=\frac{L_{PSD}}{2\cdot U_{sum}}\cdot \left[\begin{array}{c} U_{x}\;\\ U_{y} \end{array}\right].$$

Here, the positions $u_{d}$ and $v_{d}$ on the PSD can be calculated with the length of the PSD sensor $L_{PSD}$ = 10 mm and the three voltages ($U_{x}$, $U_{y}$, and $U_{sum}$) that are output by the PSD. Here, $U_{x}$ and $U_{y}$ are proportional to the laser spot’s position. The voltage $U_{sum}$ is proportional to the laser spot’s intensity.

The calculated PSD position is transformed onto the coordinate system of the screen based on camera calibration. This calibration used the pinhole model of Zhang [15] in combination with the full Brown–Conrady distortion model [18]. The process and calibration steps are explained in more detail in Section 2.2.

To compensate for misalignments of the scanner, a special method, herein denoted as ray trace shifting technique (RTST), is used. Therefore, the detection part of the system (see Figure 1b) is mounted on a translation stage (MTS25/M-Z8, Thorlabs GmbH, Munich, Germany) with a total traveling range of 25 mm, which is oriented parallel to the static beam path. This enables measurements of the projection figure at different distances between the screen and the scanner and generates additional information about the correct distance between the scanner’s pivotal point in regards to the measurement setup. It further allows measuring static vector-based as well as dynamic steering angles. The method is explained in more detail in Section 2.3.

In a final step, the sensor reading, the calibration data, and the results of the RTST are used to calculate the horizontal steering angle of the MEMS scanner. The detailed calculation is described in Section 2.4.

### 2.2. Camera Calibration

The PSD in combination with the lens must be calibrated since both elements inherently have a distortion, which would decrease the performance of the system. To compensate for this distortion, the PSD camera is calibrated with the use of Zhang’s camera model [15] and the Brown–Conrady distortion model [18] against a series of laser spot positions similar to [19]. Widely used in the field of object detection, this method applies a series of matrix multiplications to transforming the camera’s coordinate system into a real-world coordinate system.

#### 2.2.1. Calibration Process

**Figure 2:**
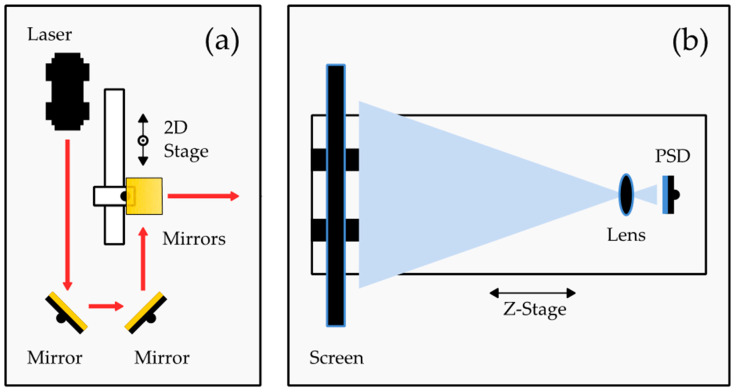
Setup for calibrating the testbench with two translation stages: (**a**) emission path; (**b**) detection path.

The measured position on the diffuser screen is calibrated against a grid of laser spots by translating a single laser spot similar to the grid of laser diodes in [19]. The setup of calibration is depicted in Figure 2.

Here, the main CW laser beam is translated with two additional mirrors, each fixed to one translation stage. The horizontal stage (Thorlabs LTS300/M) moves across a range of 30 mm, and the vertical stage moves across a range of 5 mm (Thorlabs PT1/M). The laser beam is translated in the X and Y directions along with the measurement range. When setting up the system, it was carefully ensured that the X and Y stages are aligned along the axes of the setup, which are set by the baseplate. For a detailed calibration, a rectangular grid of 29 × 7 measurement positions with a spacing of 1 mm was chosen. The PSD’s signal was captured and processed according to Equation (1) for every position of the X and Y stages. The Z-stage of the PSD was not moved during the process. 

#### 2.2.2. Calibration Arithmetic

The evaluation of the taken measurements is based on the pinhole model for cameras. A simplified and adapted version for this setup from [15] is expressed in Equation (2).

(2) $$s\cdot \left[\;\begin{array}{c} X\\ Y\\ 1 \end{array}\right]=\left[\begin{array}{ccc} \alpha & \gamma & u_{0}\\ 0 & \beta & v_{0}\\ 0 & 0 & 1 \end{array}\right]\cdot \left[\begin{array}{ccc} \cos\left(\delta \right) & \sin\left(\delta \right) & t_{x}\\ -\sin\left(\delta \right) & \cos\left(\delta \right) & t_{y}\\ 0 & 0 & 1 \end{array}\right]\cdot \left[\;\begin{array}{c} u\\ v\\ 1 \end{array}\right]$$

The parameters u and v are the horizontal and vertical undistorted coordinates on the PSD sensor surface, which become transformed to the coordinates on the screen X and Y. The first matrix on the right side of the equation is the so-called intrinsic camera matrix, which adjusts for translations $u_{0}$, $v_{0}$, scaling α and β, and skew γ. The second matrix is a simplified extrinsic matrix, which describes the positioning of the scene in regards to the camera in the form of translation $t_{x}$ and $t_{y}$ and the rotation δ around the *Z*-axis. This expression of the extrinsic matrix is a simplification that can be made due to the fixed distance and orientation between the PSD, the lens, and the projection plane. The parameter *s* is a scaling factor.

An extension to the model, which is explained in [18], suggests a series of terms to model the distortion of the lens (Equation (3)):

(3) $$\left[\begin{array}{c} u_{d}\\ v_{d} \end{array}\right]=\left[\begin{array}{c} u_{u}+\left(u_{u}-u_{0}\right)\left(k_{1}r^{2}+k_{2}r^{4}\right)+\left(p_{1}\left(r^{2}+2{\left(u_{u}-u_{o}\right)}^{2}\right)+2p_{2}\left(u_{u}-u_{0}\right)\left(v_{u}-v_{0}\right)\right)\\ v_{u}+\left(v_{u}-v_{0}\right)\left(k_{1}r^{2}+k_{2}r^{4}\right)+\left(p_{2}\left(r^{2}+2{\left(v_{u}-v_{o}\right)}^{2}\right)+2p_{1}\left(u_{u}-u_{0}\right)\left(v_{u}-v_{0}\right)\right) \end{array}\right].$$

Here, $k_{1}$, $k_{2},$ and $p_{1}$*,* $p_{2}$ are constants, and r is the length of the radius from the sensor’s origin $u_{o}$ and $v_{o}$ to the image of the detected laser spot u, v. This model calculates the distorted image from an undistorted image.

The measured positions of captured points described in Section 2.2.1 are optimized against the set positions of the calibration stages with a least-square approach for the *X* and *Y* directions simultaneously. The resulting parameters from Equations (2) and (3)**.** create a full description of the transformation from the PSD to the screen coordinate system. This is used to convert the PSD position to the desired screen position for the measurement. 

#### 2.2.3. Calibration Results

**Figure 3:**
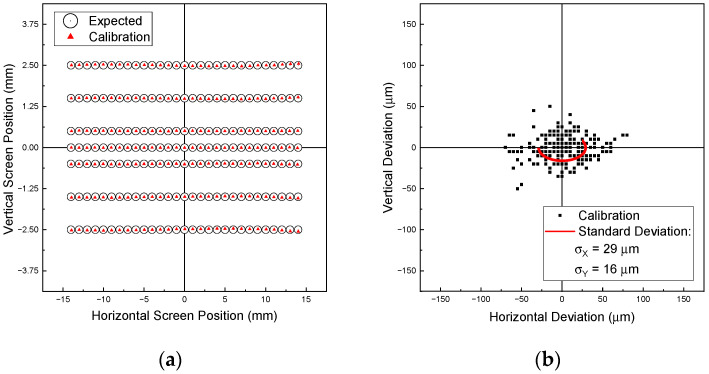
(**a**) Calibrated position on the screen (red) and the set pattern (black); (**b**) Deviations of the set values and the screen calibration (black) and the resulting standard deviation (red).

Figure 3 shows the results of the calibration. Figure 3a shows the set positions (black) and the measured positions by the system using the calibrated camera model from Section 2.2.2 (red). The deviations are depicted in Figure 3b.

Figure 3a qualitatively shows good agreement of the set and measured data over the entire range of the screen in both directions. The largest deviations occur at positive horizontal screen positions for vertical values far away from the center. A quantitative statement about the quality of the method can be derived from Figure 3b. Here, it can be seen that the maximum deviations are ca. ± 80 μm in horizontal and ca. ± 50 μm in the vertical direction, respectively. The standard deviations are calculated to be ±29 μm and ±16 μm, respectively.

### 2.3. Ray Trace Shifting Technique (RTST)

The setup is designed in such a way that the screen calibration must be performed only once. The accurate position and mounting angle of the scanner are not relevant since a unique technique is used to compensate for these effects. This marks the main advantage and benefit of the system because it reduces alignment effort and speeds up the process. The ray trace shifting technique (RTST) enables the measurement of the static steering angles of each scanner. Woong Yoo et al. [14] used a similar kind of shifting to measure the distance accurately. They used a stage with six degrees of freedom to align each scanner in front of the PSD sensor. The herein-proposed technique, however, is a wide-ranging extension that removes the need to mount each mirror precisely with optical stages. The following paragraphs cover the detailed process of the RTST.

**Figure 4:**
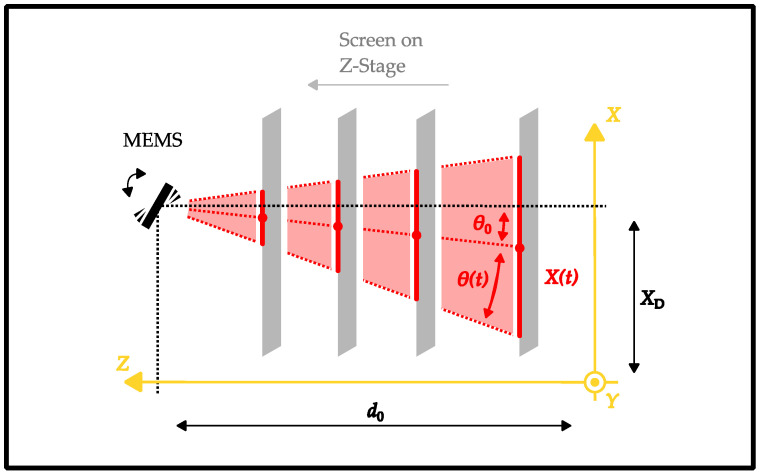
Concept of the ray trace shifting technique (RTST).

After the first measurement of the projection figure on the screen, the translation stage is shifted to measure the same oscillation at different distances. This changes the position on the screen and generates a scaled projection figure, whose extent is proportionally larger with increasing distance. The concept of the RTST is illustrated in Figure 4.

As all captures for each stage position are triggered at the same point in time after starting up the scanner, measured positions X(t) with the same time stamps can be used to calculate a vector of the steered laser ray. For this, linear regression is used (e.g., red dotted lines). By calculating the inverse tangent (arctangent) of the slope of any vector, a steering angle can be derived. This method is called “vector-based” from here on. The most significant merit is the static orientation angle $\theta_{0}$, which describes the rotational mounting errors of the scanner. 

Furthermore, the RTST provides the means to calculate the positioning error of the scanner compared to the system’s coordinates (yellow). Based on the vector calculation of each ray, the intersection of each measurement vector over time with the static steering ray can be evaluated for an oscillating scanner. The intersection distance $d_{0}$ is equal to the distance between the scanner’s pivotal point and the zero position of the stage. The intersection offset $X_{D}$ is equal to the difference between the detection axis and the scanning axis in the horizontal plane. Both parameters are retrieved by calculating the mean value across the fully captured time over several oscillations. They provide vital information on the orientation of the scanner in the setup.

When using the RTST as described here with a calibrated screen, we can compensate for misalignments of the scanners directly during the measurement process. This greatly reduces the calibration effort and thus the required time. Furthermore, it allows for dynamic steering-angle measurements.

### 2.4. Trigonometric Calculation of the Steering Angle

The measurement of the dynamic position of the laser spot on the screen and the results of the RTST are used to dynamically calculate the steering angle of the scanner dynamically. It is based on the trigonometric relationship between the steered ray, the position, and the orientation of the screen. The horizontal steering angle of the scanner with this trigonometric method is calculated as follows (Equation (4))

(4) $$\theta \left(t\right)=-\arctan\left(\frac{X\left(t\right)-X_{D}}{d_{0}-z}\right)-\theta_{0}.$$

Here, *X*(*t*) is the time-dependent horizontal laser-spot position on the screen, while $X_{D}$ is the horizontal offset of the detector axis compared to the scanner axis. The parameter $d_{0}$ describes the distance between the scanner’s pivotal point and the screen’s surface for a stage position of *Z* = 0. It is therefore also equal to the maximum distance between the scanner and the screen. The angle $\theta_{0}$ is the offset steering angle for the non-oscillating scanner in the horizontal direction. The parameters $\theta_{0}$, $d_{0}$, and $X_{D}$ result from the ray trace shifting technique described in Section 2.3.

## 3. Results

This chapter describes how the proposed setup is validated using two different approaches. Firstly, the system is validated with a static mirror that is rotated with an additional stage in front of the screen. In a second validation step, an oscillating MEMS scanner is characterized in regard to its FoV for three different excitation amplitudes.

### 3.1. Static System Validation

To verify the quality of the system’s measurements, a mirror is mounted on a rotation stage (Thorlabs PR01/M) and used on the emitting side of the setup. The static validation process with rotational stages is similar to what had been performed in the past [12].

A series of rotation angles in a total range of ca. 50° are set in the horizontal direction with a maximum error of ca. ± 0.2°. The detection side of the setup (PSD camera and the screen) is moved backward at each set angle along the *Z*-axis to 17 different distances that are equally spaced over the total range of 25 mm. This allows the measurement of a vector and results in accurate information on the mirror positioning, as described in Section 2.3. These results are furthermore used to calculate the steering angle based on Equation (4). The results of the vector-based angles, the trigonometric-based steering angles, and the set angles of the stage are referred to the first angle of each data set for a comparison of the relative angle that is equal to an FoV measurement.

**Figure 5:**
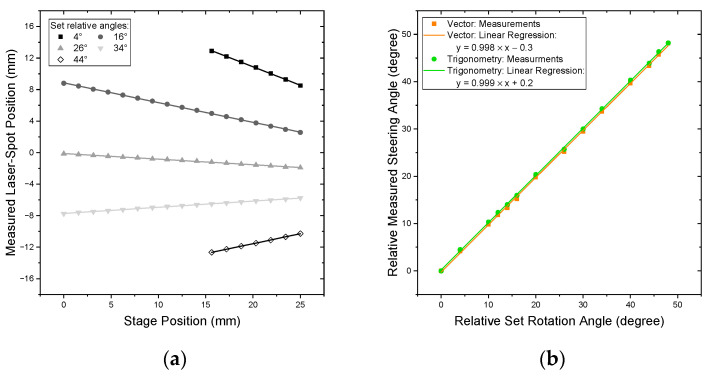
(**a**) Measured horizontal laser spot positions on the screen vs. the *Z*-axis stage position for several relative set rotation angles; a linear regression for each depicted set angle is included; (**b**) measured angles vs. the set angle including linear regressions for both the vector and trigonometric method of calculation.

The results of the vector-based static angle measurement from the RTST are depicted in Figure 5a. Here, the measured horizontal laser-spot positions on the screen are shown against the stage position. Various set rotation angles are visible with linear regressions. Figure 5b shows the resulting relative steering angles of the vector-based and the mean trigonometric-based calculations across all distances in comparison to the set values. Since no absolute angle can be set with this validation method, the relative steering angles are compared.

Figure 5a shows a linear behavior of the horizontal measured laser spot position in relation to the *Z*-axis of the stage position. The difference in the number of measurement points per set stage angle is caused by the limited screen area calibrated. For *Z*-stage positions further away from the mirror, the laser beam exceeds the system’s bounds. This is the case especially for laser beams steered with high angles since they are positioned at the edge of the measurement range of the screen.

The vector-based method of the RTST proposed in Section 2.3 is shown in the form of the linear regressions in Figure 5a. Qualitatively, a good fit is achieved. The remaining residuals of the vector-based method are in the magnitude of the calibration error of approx. 50 μm. The slopes of these regressions are depicted in Figure 5b.

As described in Section 2.3 and Section 2.4, vector-based and trigonometric-based steering angles are calculated from captured data. Figure 5b evaluates the relationship between the relative set and the relative measured steering angles. For both calculation methods, the slope of linear regression is almost 1. The remaining maximum absolute residuals are 0.44° for the trigonometric approach and 0.53° for the vector approach, respectively. These deviations depict stochastic errors that are in the same order of magnitude as the expected error of the reference of ca. ±0.4° (=2 × ±0.2°). Finally, the linear regressions result in two offset values of +0.2° and −0.3°, respectively. They can be read as the absolute deviation between the reference and the measurement.

The evaluation shows that a relative steering angle can be correctly derived with an uncertainty of 0.2° for the trigonometric approach and 0.3° for the vector approach. For the measurement of an FoV at 45°, these deviations are below 1%.

### 3.2. Proof of Concept with a Dynamic MEMS Scanner

As a final verification of the system’s capabilities, a MEMS scanner from Blickfeld GmbH with a set driving amplitude is operated in a constant sinusoidal mode at its eigenfrequency of ca. 280 Hz. The full oscillation is captured with the proposed system, and the steering angles are calculated according to the vector-based and trigonometric-based methods. The FoV of this measurement is then compared against a reference. This comparison shows that a dynamic measurement of the FoV gives the same results as with a static measurement, which was shown to be correct in the previous Section 3.1.

For reference, the scanner is characterized by measuring the full FoV on a wall with a deflected red laser, as has also been performed by Fujita et al. [8]. The wall is set to be 2.05 m apart from the scanner and the length of the projection figure is measured with an industrial metal ruler to calculate the full FoV via means of the tangent. When calculating with a measurement uncertainty of 0.5 cm as a worst-case assumption, a theoretical FoV uncertainty of ca. 0.2° is expected, which is below the required 1% value for all scanner settings.

**Figure 6:**
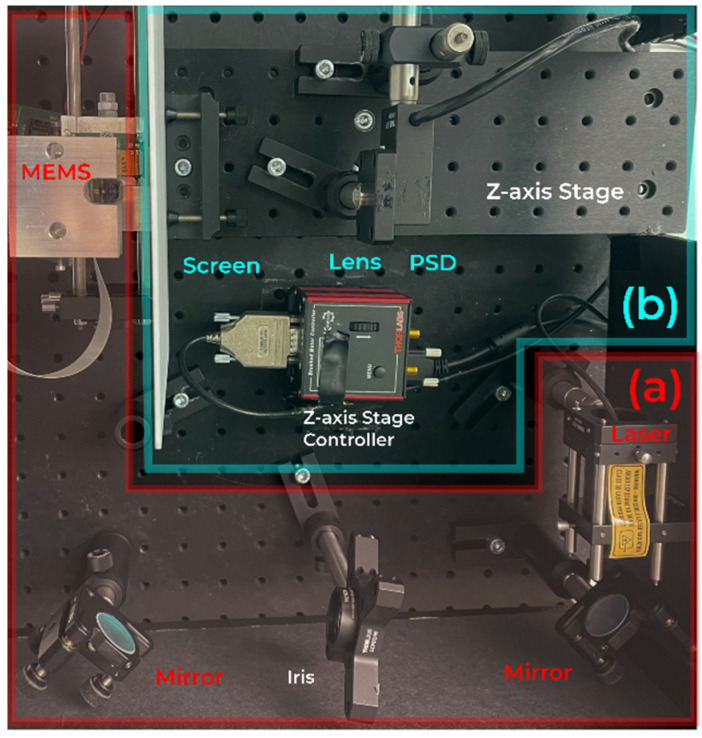
Picture of the main configuration of the setup to characterize a MEMS scanner. The overlay shows the used components in the laser part (**a**) and the detection part (**b**).

The same scanner is then placed in the proposed main configuration according to Figure 1. It is shown in Figure 6. During this measurement, the maximum measured FoV over several oscillation periods is used. To accurately measure the RTST scanner parameters, a total of ten equally spaced positions over a maximum range of 24 mm are selected. As the captured line figure on the screen gets larger with increasing distance between the scanner and the screen, and due to the fact that the usable area of the screen is limited, only stage positions closer to the scanner can be used for larger FoVs. Hence, the actual range of sampling points in Z-direction deviates depending on the FoV. The resulting angles of the computation explained in Section 2 are compared to the reference measurement results.

**Figure 7:**
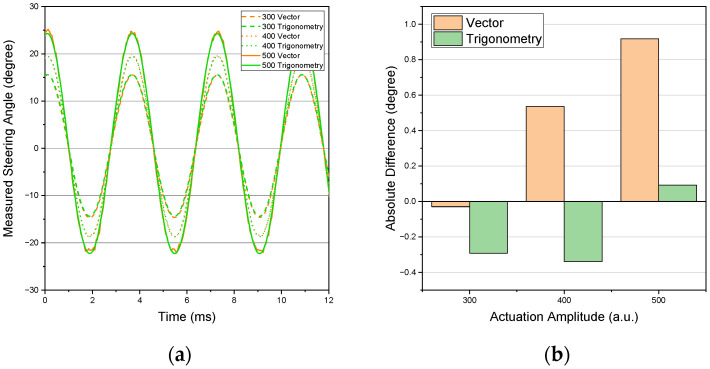
(**a**) Measured steering angles for three actuation amplitudes of 300, 400, and 500 a.u. calculated with the vector and the trigonometric approaches; (**b**) angular difference of the vector and trigonometric methods in comparison to the reference FoV measurement.

Figure 7a shows the measured steering angles over time of the MEMS scanner for the different actuation amplitudes with matched phases. Figure 7b displays the results of the FoV comparison with the reference measurement.

As seen in Figure 7a, the oscillation follows a sinusoidal movement with fixed amplitude and frequency. The graphs in orange are derived by using the vector method based on the RTST, while the green graphs are calculated via the using trigonometric approach. The latter was also averaged across all distances of the stage.

Curves with the same actuation amplitude but different calculation methods match each other very well. This is a quantitative sign for the correct implementation of the two different approaches to calculating the steering angle. The increased noise of the vector-based calculation (orange), especially at the maxima of the oscillations for higher actuation amplitudes, comes from the lack of sampling points during the RTST. The larger the FoV gets, the smaller the range in distance due to the limited size of the calibrated area on the screen. This was also discussed in Section 3.1. This means that only captures for stage positions closer to the scanner can be used for high FoVs, which reduces the total number of sampling positions for the RTST. Since this error would not represent the actual capabilities of the system, we examine the maximum FoV, which is less affected by this limitation.

The results of such an FoV comparison over several oscillations are depicted in Figure 7b. Here, the increasing error of the vector method with increasing actuation amplitude is evident. It is caused by the difference in sampling points as was discussed in the previous paragraph. The results of the trigonometric calculation are around −0.3° for 300 a.u. and 400 a.u. and ca. 0.1°at 500 a.u. The remaining deviation is within the expected uncertainty of the reference. In general, the calculation from Equation (4) is most sensitive to changes in the distance between the scanner and the pivotal point of the scanner. Due to the small distances of ca. 23 mm to 48 mm between the scanner and the screen, the distance can only be calculated accurately to ca. ± 1 mm.

As a summary, an overview of the results depicted in Figure 7 can be found in Table 1 below.

## 4. Discussion

This chapter analyzes the results of the three areas of this paper: the calibration, the static validation with a rotational stage, and the dynamic validation in the form of a proof of concept with an oscillating MEMS scanner.

Calibrating the measured position on the PSD sensor surface against the position on the screen with a laser on several positions resulted in a standard deviation of ±29 μm in the horizontal direction and ±16 μm in the vertical direction, respectively. The camera model of Zhang et al. [15] that was used therefore seems reasonable and well suited for the setup. Further improvements could be made with an imaging lens with a longer focal length or by using a better controlled single-mode laser that would result in a wider usable area on the screen.

To validate the proposed system, we performed two different validations. The first—a static validation—compared the measured steering angles against the set steering angles by rotating a mirror with a manual rotation stage. The linear regression of the FoVs resulted in a slope of 0.998 for the vector-based and 0.999 for the trigonometric approach, respectively. This verified the correctness of the setup. The remaining absolute deviation of the regression of +0.2° and −0.3° are systematic errors of the absolute steering angle. Considering the maximum relative angle of ca. 50°, the uncertainty of the system is less than 1%.

To show that the system is capable of measuring time-dependent steering angles—especially of MEMS scanners—with the same precision as static ones, a dynamic validation was performed. Therefore, a scanner was operated in a constant sinusoidal mode, and the total absolute FoV was compared to a reference. The vector-based calculation showed the highest deviations of up to 1° compared to the reference. For a dynamic “one-shot” measurement, the trigonometric approach is required. It resulted in a maximum absolute deviation of 0.3°. The traced oscillation was sinusoidal, as was expected. Since all three actuation amplitudes showed the same magnitude of deviation, it can be assumed that dynamic measurements in this angular range are also valid. This would also fit previous results [11], which demonstrated that dynamic measurements with movements slower than 10 mm per 100 μs are possible when using a similar PSD as with the proposed setup, since the maximum speed of this setup is less than 0.5 mm per 100 μs.

It is evident that the uncertainties for both validations are in the same magnitude of 0.3°. This is supported by the fact that two different references and validation methods were used to validate the performance of this setup.

The remaining uncertainty of the system for dynamic “one-shot” measurements (trigonometric method) of 0.3° does match the required metric of 1% for all tested FoVs statically and dynamically, respectively. For future work, to improve this further, we suggest calibrating a wider area on the screen as mentioned above. This would allow for measurements at greater distances between the scanner and the screen, which reduce the overall uncertainty.

## 5. Conclusions

This paper introduces a novel approach in dynamically measuring and characterizing large-FoV MEMS scanners, especially for LiDAR sensors. Based on a calibrated PSD camera, the steering angle of the scanner can be calculated by measuring the movement of a projected laser spot on an optical diffuser screen. An innovative ray trace shifting technique reduces the required calibration equipment and enables fast measurements. A static validation with a rotation stage resulted in a maximum uncertainty of 0.3°, while a dynamic validation with a MEMS scanner also led to a deviation of 0.3° for FoV measurements at ca. 47° compared to a reference. It was shown that this setup is capable of highly dynamic “one-shot” MEMS-scanner measurements with large steering angles and a low calibration effort with an uncertainty of less than 1%.

Further extensions can be made to also measure distortions of scanner pairs, as they are nowadays used on 3D-LiDAR applications. Future work must investigate the usage of larger FoVs and the improvement of measurement uncertainty. The FoV can be enlarged by using a different imaging lens to cover a larger area on the screen. A longer traveling stage and the capturing of additional projection figures will improve the measurement uncertainty.

## Figures and Tables

**Table 1:** Results of the dynamic validation.

Actuation Amplitude (a.u.)	Reference (Degree)	Vector (Degree)	Trigonometry (Degree)
300	30.3	30.3	30.0
400	38.6	39.1	38.2
500	46.5	47.5	46.6

## Data Availability

Data is contained within the article.
